# Too much to learn, too little to move? The impact of course load on university students’ physical wellbeing

**DOI:** 10.3389/fpubh.2026.1737339

**Published:** 2026-02-11

**Authors:** Huarong Wu, Fangrong Wu, Chunli Zhang, Yucheng Gao

**Affiliations:** 1 School of Physical Education, Changsha University of Science and Technology, Changsha, China; 2 School of Physical Education, Hunan International Economics University, Changsha, China; 3 Institute of Physical Education and Training, Capital Institute of Physical Education and Sports, Beijing, China; 4 College of Physical Education, Hunan Normal University, Changsha, China

**Keywords:** academic workload, fixed effects model, gender differences, nonlinear threshold, physical health, university physical education

## Abstract

**Objective:**

Against the background of increasing academic workload in higher education, this study examines the impact of academic course hours on college students’ objective physical health, measured by comprehensive physical fitness scores, and explores potential gender differences and nonlinear patterns.

**Methods:**

Using a three-year longitudinal panel dataset (*N* = 305, 915 person-years) from a 2020 cohort at Changsha University of Science and Technology, we applied individual fixed effects models, supported by Hausman tests, to estimate the net effect of academic workload. The dependent variable was students’ comprehensive physical fitness score, while the key independent variable was total annual academic (non-PE) course hours. Physical education (PE) course hours were included as a control variable. To identify nonlinear relationships, polynomial, logarithmic, and square root functional forms were tested, and gender-stratified analyses were conducted.

**Results:**

Academic workload significantly and negatively affected students’ physical health, with each additional academic hour associated with a 0.012-point decrease in physical fitness scores (*p* < 0.001). In contrast, PE course hours had a significant positive effect, increasing scores by 0.088 points per hour (*p* < 0.001). Gender differences were evident: the negative impact of academic workload was stronger among male students (*B* = −0.013) than among females (*B* = −0.009), and the health benefits of PE were also greater for males. Furthermore, the relationship between academic workload and physical health exhibited a clear nonlinear pattern. Physical health improved at low workload levels (<547 h), declined at moderate to high levels (547–1,087 h), and showed a slight marginal rebound at very high levels (>1,087 h). Among all tested specifications, the cubic model provided the best fit based on AIC and BIC criteria.

**Conclusion:**

Academic workload exerts a significant, gender-differentiated, and nonlinear influence on college students’ physical health. Universities should maintain academic demands within an optimal range and ensure a balanced allocation between academic and physical education coursework. Targeted interventions, particularly for male students, are recommended to mitigate the adverse health effects of excessive academic burden.

## 1 Introduction

In recent decades, higher education worldwide has experienced unprecedented expansion, with university enrollment rates rising sharply and the number of students entering higher education reaching record highs (1, 2). While this trend has broadened access to educational opportunities, it has also intensified academic competition—students now face heightened pressures in terms of academic achievement, employability, and postgraduate admissions (3). To cope with this competitive climate, many universities have increased course loads, assigned more frequent assessments, and incorporated supplementary tasks such as peer evaluations and additional readings into their teaching practices (4, 5). Although intended to enhance academic engagement, these practices have inadvertently increased students’ course load, potentially posing risks to their physical health—an essential foundation for sustaining learning efficiency and academic success.

Understanding the relationship between course load and students’ physical health is therefore of both theoretical and practical importance, with implications for the quality of higher education and students’ sustainable development. However, systematic research specifically examining this relationship remains scarce. To fill this gap, the present study draws on the broader literature on academic stress as a conceptual reference (6). For instance, a cross-sectional study by Asensio-Martinez et al. (7) reported a significant negative association between academic burden and students’ self-perceived health status, suggesting that excessive academic demands may undermine subjective wellbeing. Beyond psychological impacts, academic stress has also been linked to physiological outcomes. Casuso-Holgado et al. (8) found a significant positive relationship between academic stress and physical fatigue in university students, while Ekpenyong et al. (9) observed a higher prevalence of musculoskeletal disorders among students under prolonged academic stress, with female students being disproportionately affected. Similarly, Hystad et al. (10) reported a positive association between academic stress and self-reported health problems, and Vuletić and Erdeši (11) further confirmed that academic stress adversely affects multiple dimensions of health, with female students experiencing greater health deterioration. From a psychosocial perspective, MacGeorge et al. (12) highlighted that academic stress is associated not only with depression and physical illness but may also erode social support networks, thereby exacerbating health risks. Notably, social support was found to have a significant buffering effect.

While the existing literature provides compelling evidence of a negative relationship between academic stress and student health, three key limitations persist. First, conceptually, most studies focus on the broad notion of “academic stress,” with limited attention to course load as a distinct educational variable that reflects the structural features of teaching systems and task design. Second, in terms of measurement, reliance on self-reported health introduces potential biases. Third, methodologically, most studies are cross-sectional, limiting the ability to infer causal relationships.

To address these limitations, this study utilizes a three-wave panel dataset (*N* = 305, 915 person-years) from a 2020 cohort at a Chinese university to examine the relationship between course load and students’ physical health. The dependent variable is students’ comprehensive physical fitness score based on the National Student Physical Health Standard, providing an objective measure of health. A fixed effects panel model is employed to control for unobserved individual heterogeneity. Additionally, the study examines gender differences and tests for nonlinear effects.

This study contributes to the literature in several ways:

1. Conceptual focus: It refines the analytical focus from the general notion of “academic stress” to the more institutionally specific concept of “course load,” thereby enhancing the explanatory power regarding educational governance;
2. Measurement improvement: It employs objective physical fitness scores to reduce potential bias associated with self-reported measures;
3. Methodological advancement: By leveraging panel data and fixed effects modeling, the study allows for quasi-causal interpretation and identification of threshold and nonlinear effects;
4. Practical implications: The findings offer actionable insights for optimizing curriculum design, balancing academic and physical education, and supporting the physical and mental health of university students.

## 2 Research design

### 2.1 Data source and processing

This study was conducted at Changsha University of Science and Technology, which was selected primarily because of the institutional completeness and accessibility of its administrative records. The university maintains standardized and longitudinally consistent data on students’ academic course hours and physical education participation, and strictly implements the National Student Physical Health Standard, ensuring the reliability and comparability of physical fitness measurements across years. This study adopts a random cluster sampling approach, in which one college was randomly selected from all colleges within Changsha University of Science and Technology to serve as the research sample. The selected unit was the School of Physics and Electronic Science. To ensure both the recency and completeness of the data, we focused on students from the 2020 cohort, the most recent class with complete four-year follow-up data.

The initial sample comprised 403 students from the 2020 cohort. To maintain data integrity, we excluded two categories of individuals: students officially exempted from the physical fitness test due to injury, illness, or other special circumstances; students with missing or unrecorded physical test scores.

After data cleaning, the final analytical sample included 305 students with complete panel data. Given that fourth-year courses are primarily composed of weekly practical training, making it difficult to accurately calculate course hours, data from the senior year (2023) was excluded from the analysis.

Thus, the study covers a three-year period from 2020 to 2022, corresponding to students’ freshman through junior years, yielding a total of 915 observations (305 students across 3 academic years).

### 2.2 Variable description

The dependent variable in this study is students’ physical health, operationalized through the comprehensive score from the National Student Physical Health Standard, ranging from 0 to 100. This standardized fitness test offers an objective assessment of individuals’ physical function and athletic performance, and is thus a widely accepted indicator of physical health (13, 14).

The fitness test components differ by gender:

Male students are assessed on: 50-meter sprint, 1,000-meter run, height, weight, body mass index (BMI), pull-ups, standing long jump, vital capacity, and sit-and-reach flexibility.

Female students are assessed on: 50-meter sprint, 800-meter run, height, weight, BMI, one-minute sit-ups, standing long jump, vital capacity, and sit-and-reach flexibility.

The weighted formula for calculating the comprehensive physical fitness score is as follows:

BMI × 15% + Vital Capacity × 15% + 50 m Sprint × 20% + Sit-and-Reach × 10% + Standing Long Jump × 10% + (Pull-ups or Sit-ups) × 10% + (1,000 m/800 m Run) × 20%.

Due to gender-specific scoring standards for each component (e.g., 50-meter sprint, standing long jump, vital capacity, sit-ups/pull-ups), the detailed conversion tables are not presented in the main text but are available in the Supplementary Table S1.

The primary independent variable is course load, defined as the total number of academic (non-PE) course hours per academic year, measured in class hours (1 class hour = 45 min). To estimate the net effect of academic workload on physical health, physical education courses are excluded from this calculation.

PE course hours (also measured annually in class hours) are included as a control variable to account for the direct influence of physical education on students’ physical fitness outcomes.

Unless otherwise noted, all variables are constructed on an academic-year basis.

### 2.3 Analytical method

This study adopts the Fixed Effects (FE) Model as the primary analytical approach. The fixed effects model is a panel data regression technique that controls for unobservable, time-invariant individual heterogeneity, thereby reducing estimation bias and enhancing the reliability of the results. It is particularly suited for examining how within-individual changes in explanatory variables over time affect the outcome variable, while accounting for stable individual-level characteristics.

In our context, employing a fixed effects model helps control for inherent personal traits—such as baseline physical fitness—allowing for a more precise estimation of the quasi-causal effect of course load on physical health.

To ensure the robustness of our findings, we conducted the following additional analyses:

Hausman test: this test was used to determine whether the fixed effects model is more appropriate than the random effects model, based on the consistency and efficiency of the estimators.
Heterogeneity analysis: we stratified the sample by gender identity and conducted separate regressions for students identifying as male and students identifying as female to assess whether the observed effects vary across subgroups.
Nonlinear relationship testing: we further explored whether nonlinear functional forms provide a better fit for modeling the relationship between course load and students’ physical health.

## 3 Empirical analysis

### 3.1 Descriptive statistics of variables

To examine the distribution of key variables in this study, we conducted descriptive statistical analysis. The results are presented in Table 1.

**Table 1:** Descriptive statistics of the sample.

Variable	Observations	Mean	Std. Dev.	Min	Max
Gender (1 = Male, 0 = Female)	915	0.770	0.421	0	1
PE course hours (per year)	915	40.000	28.265	0	60
Course load (course hours per year)	915	767.331	119.842	424	1,326
Physical fitness score	915	67.498	8.538	30.3	93.2
Age at baseline (years)	305	18.732	0.643	17	21

All variables are measured on an academic-year basis unless otherwise specified.

The sample includes students of all genders, with men comprising 77.05% of all observations—an expected distribution given the engineering-oriented discipline of the sampled college. In terms of physical education (PE) course hours, the average is 40 class hours per academic year, with a minimum of 0 and a maximum of 60. For academic course load, the average is 767.331 class hours per year, with a range from 424 to 1,326. The average physical fitness score, based on the *National Student Physical Health Standard*, is 67.498, with a minimum of 30.3 and a maximum of 93.2.

### 3.2 Fixed effects model

To account for unobserved, time-invariant individual heterogeneity—such as students’ innate physical abilities or intrinsic interest in exercise—this study employs a Fixed Effects (FE) Model. Given the potential for heteroscedasticity in panel data, we further apply clustered robust standard errors at the individual level to adjust the standard errors and enhance the robustness of the estimates. The regression results are summarized in Table 2.

**Table 2:** Fixed effects regression results.

Variable	Coefficient	Std. Error	*t*-value
PE course hours	0.088***	(0.006)	14.23
Course load	−0.012***	(0.002)	−7.76

*p < 0.05, **p < 0.01, ***p < 0.001. Standard errors clustered at the individual level are reported in parentheses.

The results indicate a significant positive effect of physical education (PE) course hours on students’ physical health. Specifically, for each additional PE class hour per academic year, the comprehensive physical fitness score increases by an average of 0.088 points (*p* < 0.001).

In contrast, course load—measured by academic course hours per year—shows a significant negative effect on physical health. Each additional academic course hour is associated with an average decrease of 0.012 points in the physical fitness score (*p* < 0.001).

These findings underscore the contrasting impacts of academic and physical education workloads on students’ physical health, supporting the need to balance curriculum structure to promote student wellbeing.

### 3.3 Robustness checks

#### 3.3.1 Hausman test

To validate the appropriateness of our model choice, we conducted a Hausman test, which compares the estimates from the Fixed Effects (FE) and Random Effects (RE) models. The test evaluates whether the regressors are correlated with unobserved individual effects. The null hypothesis (H₀) assumes that the random effects estimator is consistent and efficient, while the alternative hypothesis (H^a^) posits that the fixed effects estimator is more appropriate due to such correlation. The results are presented in Table 3.

**Table 3:** Results of Hausman test.

Variable	FE estimate	RE estimate	Difference (FE − RE)	Std. Error
PE Course Hours	0.088***	0.086***	0.001	—
Course load	−0.012***	−0.011***	−0.001	0.000

*p < 0.05, **p < 0.01, ***p < 0.001. Standard errors are reported in parentheses. “—” indicates standard error not reported.

The Hausman test yields a *p*-value of 0.020, which is below the conventional 0.05 threshold. Therefore, we reject the null hypothesis, indicating that the assumptions underlying the random effects model do not hold. This result supports the adoption of the fixed effects model as the more consistent and reliable estimator for our analysis.

#### 3.3.2 Heterogeneity analysis

To further examine whether the relationship between course load and physical health varies by gender, this study conducts fixed-effects regression analyses separately for male and female samples. The results are presented in Table 4.

**Table 4:** Fixed-effects regression results by gender group.

Variables	Male	Female
Physical education hours	0.094***	0.068***
Course load (hours)	−0.013***	−0.009***
N (observations)	705	210
N (groups)	235	70
*R* ^2^	0.265	0.209

Standard errors in parentheses. *p < 0.05, **p < 0.01, ***p < 0.001.

**Figure 1:**
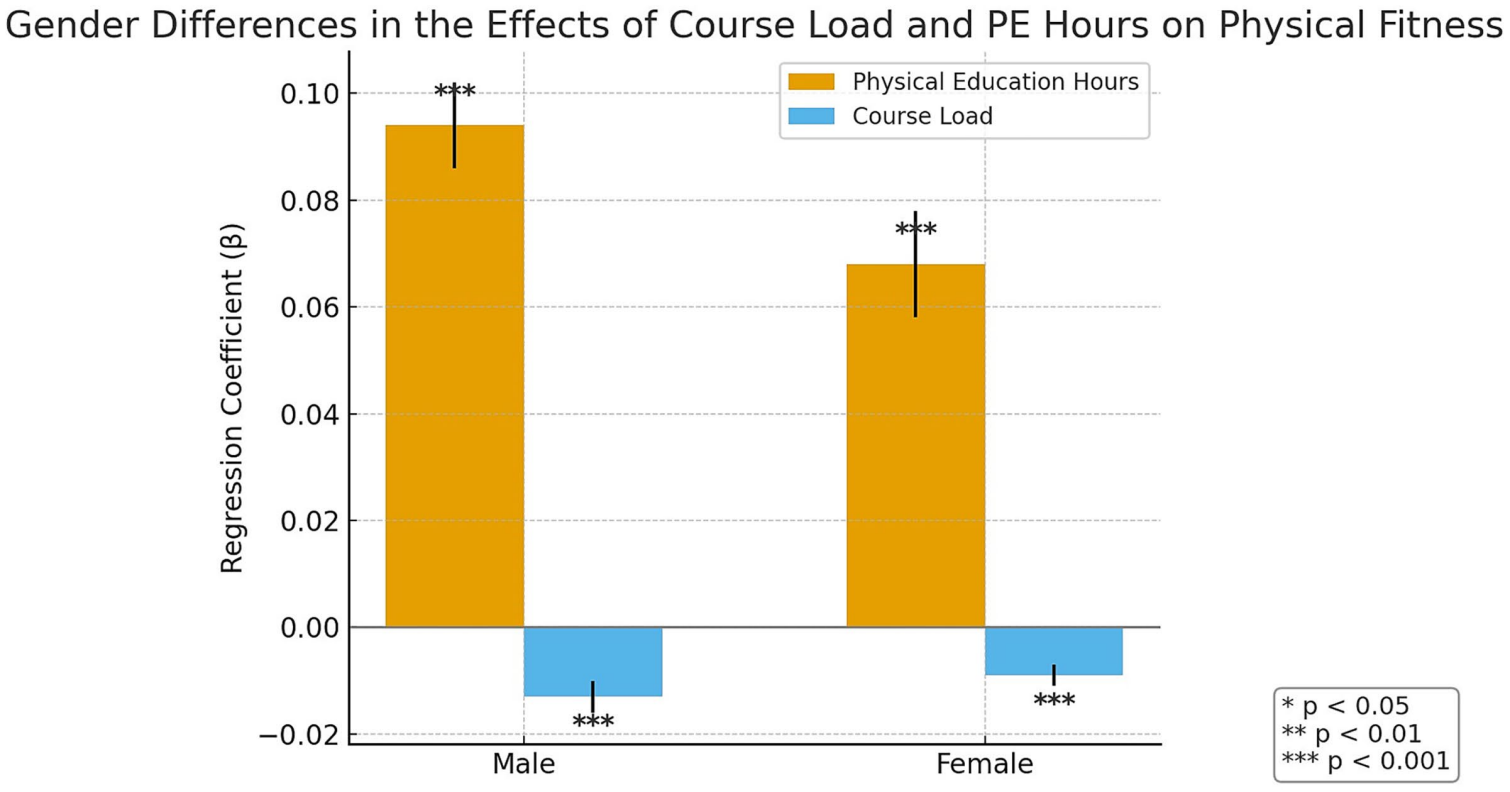
Fixed-effects regression results by gender.

To enhance clarity and objectivity, we visualized the regression results, as shown in Figure 1.

As illustrated in Figure 1, the number of physical education class hours exerts a stronger positive effect on male students’ physical health (*B* = 0.094, *p* < 0.001). Although the effect size is smaller for female students (*B* = 0.068, *p* < 0.001), it remains statistically significant. Likewise, course load imposes a more pronounced negative effect on male students’ physical health (*B* = −0.013, *p* < 0.001). Specifically, each additional hour of course load is associated with an average decrease of 0.013 points in male students’ physical fitness scores, compared to a 0.009-point decrease for female students.

#### 3.3.3 Test for nonlinear relationship

The fixed-effects model assumes a linear relationship between independent and dependent variables in conditional expectations. However, existing research suggests that the relationship between stress and health is nonlinear, as moderate stress can enhance physiological adaptation and behavioral regulation, while excessive stress may cause harm (15). Therefore, the relationship between college students’ course load and physical health may also exhibit a nonlinear pattern.

To investigate this possibility, we applied several common nonlinear transformations to the independent variable—course load. Specifically, we considered four types of transformations: quadratic, cubic, logarithmic, and square root. Polynomial transformations are used to capture curvilinear relationships; the logarithmic transformation aims to reflect proportional effects; and the square root transformation corresponds to diminishing marginal effects.

To evaluate and compare the model performance under each specification, we computed the Akaike Information Criterion (AIC) and the Bayesian Information Criterion (BIC) for each model. These two criteria are widely used in quantitative data analysis to assess model fit and complexity. Both favor models with lower values, although AIC places greater emphasis on predictive accuracy, whereas BIC favors model simplicity and interpretability.

As shown in Table 5, the baseline model yields the highest AIC and BIC values, indicating that the relationship between course load and physical health is unlikely to be linear. Among all specifications, the cubic model yields the lowest AIC and BIC values, suggesting that it provides the best approximation of the underlying nonlinear relationship between course load and physical health.

**Table 5:** Comparison of regression models.

Model	df	AIC	BIC	*N*
Baseline model	3	5,013	5,027	915
Quadratic model	3	4,998	5,013	915
Cubic model	4	4,992	5,011	915
Logarithmic model	3	5,007	5,022	915
Square root model	3	5,007	5,024	915

**Figure 2:**
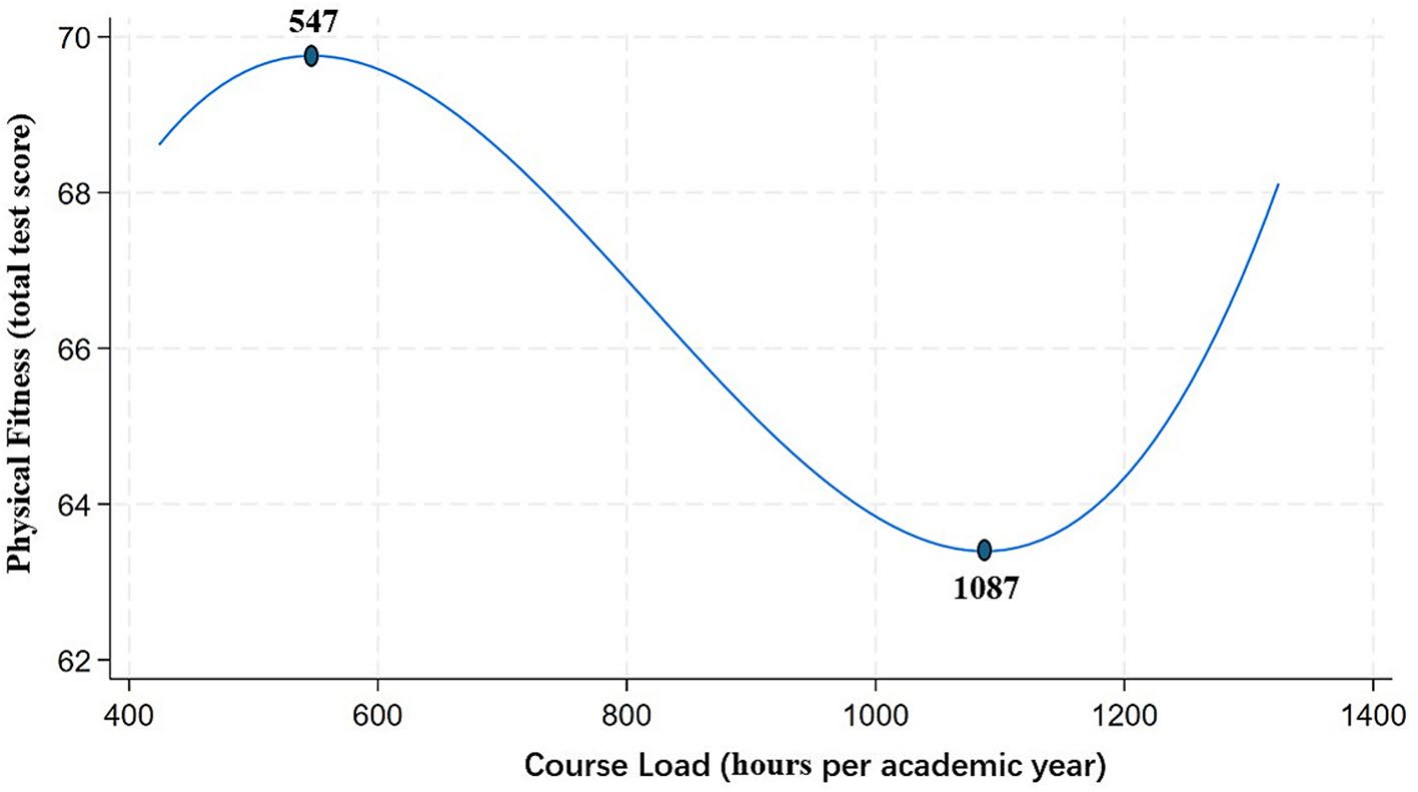
Nonlinear relationship between course load and physical health among college students.

To objectively illustrate the regression results of the cubic transformation model, we graphically represented the estimated relationship, as shown in Figure 2. In constructing this figure, we controlled for potential confounding variables by holding the number of physical education hours constant at its sample mean (40 h), and plotted only the partial relationship between course load and physical health.

The curve clearly reveals a significant nonlinear association between course load and physical health. At relatively low levels of course load (specifically, below 547 h), increases in academic load are positively associated with physical health, suggesting that a moderate academic workload may contribute to improved physical wellbeing. However, beyond this threshold (i.e., above 547 h), the positive effect diminishes and gradually reverses, with physical health beginning to decline as course load increases. Interestingly, when course load reaches an extremely high level (greater than 1,087 h—a range that includes less than 3% of the sample), the marginal effect becomes positive again.

## 4 Discussion

### 4.1 Main findings and academic contributions

Drawing on three academic years of panel data from Changsha University of Science and Technology, this study investigates the relationship between course load and physical health among university students. It conceptually substitutes the traditionally psychological construct of “academic stress” with institutionalized educational variables and employs fixed-effects modeling to approximate causal inference. This research design contributes methodological and conceptual innovations, positioning the study uniquely within the academic literature.

The analysis yields four key findings:

First, overall course load significantly undermines students’ physical health. Controlling for time-invariant individual characteristics, each additional hour of academic coursework is associated with an average decrease of approximately 0.012 points in composite physical fitness scores.

Second, physical education hours exert a significantly positive effect. Each additional hour of physical education corresponds to an average increase of about 0.088 points in physical test scores, indicating that formal physical education remains a critical institutional component in maintaining and enhancing student health in higher education contexts.

Third, the relationship between course load and physical health is distinctly nonlinear. Both model results and graphical analyses reveal a three-stage pattern: in the lower range of course load (<547 h), academic engagement appears to support physical development; in the middle-to-high range (547–1,087 h), physical health declines markedly; and at extremely high levels of course load (>1,087 h), a slight rebound in physical health is observed. However, this latter finding is based on a small subsample (less than 3%) and should be interpreted with caution.

Fourth, significant gender differences are observed. Male students are more negatively affected by course load, with a larger coefficient (−0.013) compared to females (−0.009), and benefit more from physical education in terms of improved physical fitness.

In terms of academic contributions, this study advances the literature in three main areas:

First, conceptual innovation. By introducing “course load” as a structural alternative to the vague notion of “academic stress,” the analysis shifts from subjective individual experiences to institutional determinants, emphasizing the role of educational systems and teaching practices in shaping health outcomes.

Second, measurement innovation. Unlike previous studies that predominantly rely on self-reported health measures, this study employs nationally standardized physical fitness test scores, enhancing measurement accuracy and the credibility of empirical results.

Third, methodological innovation. The use of individual fixed-effects models allows for the control of unobserved heterogeneity—such as inherent physical ability and sports interest—and, through cubic polynomial modeling, captures complex nonlinear dynamics. This approach overcomes the common limitations of cross-sectional studies, such as weak causal identification and oversimplified model structures.

In summary, this study provides robust empirical evidence of a complex and nonlinear relationship between institutional academic burden and university students’ physical health. The findings offer valuable policy insights for curriculum design and underscore the importance of achieving a sustainable balance between academic development and student wellbeing.

### 4.2 Nonlinear relationship between course load and physical health

One of the key findings of this study is the significant nonlinear relationship between course load and physical health among university students. By incorporating polynomial transformations into the fixed-effects regression model, we find that the cubic specification achieves the lowest AIC and BIC values, outperforming linear, quadratic, and logarithmic models. This suggests that the association between course load and physical fitness follows a complex curvilinear trajectory.

Figure 2 provides visual evidence of this pattern: when course load is relatively low (i.e., below approximately 547 h), physical fitness scores tend to rise with increasing coursework, indicating a potentially beneficial effect of moderate academic engagement. However, in the mid-to-high course load range (547–1,087 h), students’ physical health declines significantly. Beyond 1,087 h, the curve displays a slight upward trend, though this finding should be interpreted with caution, as observations in this range account for less than 3% of the sample (16).

This inverted U-shaped relationship can be understood through the lens of the Stress Arousal Reappraisal (SAR) theory, which posits that when individuals perceive that their personal resources are sufficient to meet or exceed external demands, they exhibit a challenge-oriented stress response, leading to enhanced performance. Conversely, when perceived demands outweigh available resources, individuals experience a threat-oriented stress response, resulting in physiological decline (17, 18). The foundational work of Selye (19) further distinguishes between eustress (beneficial stress) and distress (harmful stress), which produce divergent outcomes.

Mechanistically, several studies support the positive impact of moderate academic load. Bosshard et al. (20) demonstrated that moderate stress enhances self-management and motivational regulation, while De la Fuente et al. (21) found that students experiencing manageable academic stress are more likely to adopt proactive time management and self-regulation strategies to maintain equilibrium between study and life demands. These findings help explain the positive health effects observed in the low-load range.

In contrast, the health decline observed in the mid-to-high load range may result from both behavioral and physiological mechanisms. On the behavioral side, Asensio-Martínez et al. (7) report that academic stress often leads to unhealthy habits such as excessive caffeine consumption and high-calorie diets, which negatively affect physical health. Physiologically, Largo-Wight et al. (22) find that sustained academic overload may suppress immune system function and contribute to physical exhaustion through reduced physical activity and sleep deprivation.

Taken together, these findings point to an inverted U-shaped association between course load and physical health. Moderate academic demands appear to stimulate positive stress responses that promote health, while excessive academic burden transforms into harmful stress, weakening students’ physiological capacity. This contributes to theoretical understandings of the academic stress–health nexus and provides an empirical foundation for university curriculum reform. It suggests that educational policymakers and administrators should work toward identifying an optimal course load range, one that balances academic rigor with students’ physical and mental wellbeing.

Finally, this finding carries broader implications for the future of global higher education. For decades, university systems around the world have largely operated under an efficiency-driven logic, prioritizing credit accumulation, curriculum density, and academic output (23). While this orientation may enhance institutional performance in the short term, it risks neglecting the physical and psychological limits of students. The nonlinear relationship identified here underscores the need for a shift toward a balance-oriented educational model, which seeks not only to improve academic performance but also to systematically protect student health through thoughtful curriculum design and workload management.

### 4.3 Gender-related differences: greater adverse impact of course load among students identifying as male

The heterogeneity analysis reveals that course load has a more substantial negative impact on the physical health of male students (see Figure 1). This result aligns with findings by Asensio-Martínez et al. (7), who similarly observed that academic stress exerts stronger detrimental effects on male students’ health.

The physiological and psychological mechanisms underlying this gender disparity merit further exploration. Existing research indicates that achievement-related psychosocial stress activates the hypothalamic–pituitary–adrenal (HPA) axis and the autonomic nervous system, thereby increasing physiological strain. Chronic exposure to such academic stress can impair cardiovascular and immune system functioning, with long-term consequences for physical health (24, 25). In contrast, female sex hormones have been shown to attenuate the responsiveness of both the sympathetic nervous system and the HPA axis, thereby buffering the physiological damage induced by stress (26).

These findings carry significant implications for higher education governance and student health promotion. They suggest that academic institutions should account for gender-based differences in physiological stress responses when formulating curricular structures and assessment policies. For example, universities could implement regular monitoring of academic stress levels and fitness indicators across gender groups, and dynamically adjust the balance between academic coursework and physical education. Additionally, targeted interventions—such as enhanced opportunities for physical recovery and access to psychological counseling—should be made available to male students, in order to mitigate the long-term health risks associated with excessive academic demands.

## 5 Limitations and future research

### 5.1 Research limitations

Despite utilizing three-year panel data and applying an individual fixed-effects model to control for unobserved individual heterogeneity, this study has several noteworthy limitations.

First, the sample lacks broad representativeness. The dataset is drawn from a single cohort of students (enrolled in 2020) from one college within Changsha University of Science and Technology. Although the study tracks these students over three academic years, the relatively small and institutionally homogeneous sample restricts the external validity of the findings. Future research should expand to include more diverse samples across different regions and types of higher education institutions to enhance the generalizability of the conclusions.

Second, there are limitations in the measurement of key variables. The current study employs total annual academic course hours as an objective proxy for course load, which captures institutional burden effectively. However, this metric does not account for subjective experiences such as perceived academic stress, exam anxiety, or workload perception. As a result, the actual intensity of academic stress may be underestimated.

Third, the identification strategy may still be subject to potential endogeneity. Although the fixed-effects model controls for time-invariant unobserved individual characteristics, it does not fully address time-varying confounders—such as students’ evolving health management behaviors, changes in lifestyle, or shifts in exercise frequency over time. Future studies could adopt more robust causal identification strategies, such as instrumental variable approaches, system Generalized Method of Moments (GMM), or regression discontinuity designs (RDD), to improve the reliability of causal inference.

### 5.2 Future research directions

By leveraging 3 years of panel data, this study empirically demonstrates a significant nonlinear relationship between course load and university students’ physical health, contributing to the understanding of the complex interaction between educational investment and health outcomes. Nonetheless, several promising avenues for future research remain:

First, expanding the scope and contextual diversity of research samples. While this study focuses on a specific institutional setting, future work should conduct longitudinal analyses across various types of higher education institutions—such as comprehensive universities, teacher-training colleges, and sports universities—as well as different geographical regions. This will enable researchers to examine the robustness of the findings and explore how factors such as campus culture, academic disciplines, and course types moderate the observed associations.

Second, improving measurement frameworks. The current analysis relies on scheduled academic hours as a proxy for course load and uses national standardized fitness test scores to assess physical health. Future studies could integrate both objective and subjective indicators—such as self-reported academic pressure, perceived workload, and psychological wellbeing metrics—to construct a more comprehensive and multidimensional assessment system.

Third, deepening the analysis of mechanisms and mediation pathways. Although this study uncovers an inverted U-shaped relationship between course load and physical health, the behavioral and psychological mechanisms driving this pattern remain underexplored. Future research could develop mediation models focusing on time-use allocation, sleep quality, levels of physical activity, and stress response dynamics to better understand the causal pathways linking academic demands to health outcomes. Such insights would inform the design of more targeted and effective intervention strategies.

## Data Availability

The raw data supporting the conclusions of this article will be made available by the authors, without undue reservation.
